# Identification of useful genes from multiple microarrays for ulcerative colitis diagnosis based on machine learning methods

**DOI:** 10.1038/s41598-022-14048-6

**Published:** 2022-06-15

**Authors:** Lin Zhang, Rui Mao, Chung Tai Lau, Wai Chak Chung, Jacky C. P. Chan, Feng Liang, Chenchen Zhao, Xuan Zhang, Zhaoxiang Bian

**Affiliations:** 1 Tianjin University of Traditional Chinese Medicine, Tianjin, China; 2 Chinese Clinical Trial Registry (Hong Kong), Hong Kong Chinese Medicine Clinical Study Centre, Chinese EQUATOR Centre, School of Chinese Medicine, Hong Kong Baptist University, Hong Kong, SAR China; 3 Department of Computer Science, HKBU Faculty of Science, Hong Kong Baptist University, Hong Kong, SAR China; 4 Oncology Department, The Second Affiliated Hospital of Tianjin University of Traditional Chinese Medicine, Tianjin, China; 5 Centre for Chinese Herbal Medicine Drug Development, Hong Kong Baptist University, Hong Kong, SAR China

**Keywords:** Genetics, Gastroenterology

## Abstract

Ulcerative colitis (UC) is a chronic relapsing inflammatory bowel disease with an increasing incidence and prevalence worldwide. The diagnosis for UC mainly relies on clinical symptoms and laboratory examinations. As some previous studies have revealed that there is an association between gene expression signature and disease severity, we thereby aim to assess whether genes can help to diagnose UC and predict its correlation with immune regulation. A total of ten eligible microarrays (including 387 UC patients and 139 healthy subjects) were included in this study, specifically with six microarrays (GSE48634, GSE6731, GSE114527, GSE13367, GSE36807, and GSE3629) in the training group and four microarrays (GSE53306, GSE87473, GSE74265, and GSE96665) in the testing group. After the data processing, we found 87 differently expressed genes. Furthermore, a total of six machine learning methods, including support vector machine, least absolute shrinkage and selection operator, random forest, gradient boosting machine, principal component analysis, and neural network were adopted to identify potentially useful genes. The synthetic minority oversampling (SMOTE) was used to adjust the imbalanced sample size for two groups (if any). Consequently, six genes were selected for model establishment. According to the receiver operating characteristic, two genes of OLFM4 and C4BPB were finally identified. The average values of area under curve for these two genes are higher than 0.8, either in the original datasets or SMOTE-adjusted datasets. Besides, these two genes also significantly correlated to six immune cells, namely Macrophages M1, Macrophages M2, Mast cells activated, Mast cells resting, Monocytes, and NK cells activated (*P*  <  0.05). OLFM4 and C4BPB may be conducive to identifying patients with UC. Further verification studies could be conducted.

## Introduction

Ulcerative colitis (UC), one type of inflammatory bowel disease (IBD, another is Crohn’s Disease), is characterized as inflammation and ulceration in the rectum and colon, which may eventually affect the whole colon if left untreated^1^. The clinical manifestation of UC mainly includes bloody diarrhea, frequent bowel movement, abdominal discomfort, pain, weight loss, fever, and fatigue^2^. Unfortunately, UC is deemed as an incurable disease despite plenty of therapeutic options available depending on the disease severity. UC patients are often suffering from alternating conditions of clinical relapse and remission that severely deteriorate their quality of life^3^. The incidence of UC is previously more prevalent in high-income countries of Europe and North America has shifted towards industrialized countries such as Asia. As a result, it has become a global refractory disease with worldwide shifting epidemiological characteristics. Previous studies have found that immune dysfunction contributes to the progression of UC^4^. Specifically, in B cells, UC patients showed an increasing percentage of CD23 B naive cells than the normal individuals, while intestinal CD11b + B Cells relieve colitis by secreting immunoglobulin A^5, 6^.

Generally, the diagnosis criteria of different UC stages are mainly based on clinical symptoms and the endoscope and biochemical examinations^7^. There are some standard methods, such as Mayo score, Ulcerative Colitis Disease Activity Index (UCDAI), etc., which were widely used to identify the remission or active stage of UC^8^. Additionally, several other factors, including IL-6, TNF-α, and hs-CRP, were also helpful for the diagnosis of UC^9^. Besides, some scholars have compared the transcriptomic data of rectum biopsy in UC patients and healthy subjects, and high heterogeneity in the gene expression was observed in the UC group^10^. Particularly, genes with positive correlations were enriched among biological processes, including inflammatory response, neutrophil chemotaxis, and immune regulation^11^. Compared to other immune diseases (e.g., HLA-B27 related to Ankylosing Spondylitis), few studies have analyzed the diagnosis or transcriptome differences between UC patients and healthy individuals^12^. Moreover, although previous studies had reported the role of genes in the diagnosis of UC, the results were not satisfactory due to the database with only two microarrays or the area under the curve (AUC) were unstable^13, 14^. Thus, it is necessary to develop a predictive model for UC diagnosis with stable AUC based on multiple microarrays.

Machine learning (ML), based on a series of complex algorithms process, is recently commonly used to identify biomarkers and to predict a wide range of diseases. For example, Random Forest (one type of ML) was used to forecast Crohn’s disease and UC with higher prediction accuracy (more than 90%), even exceeding the traditional prediction model^15^. Among various ML methods, Support Vector Machine (SVM) has advantages in the diagnosis research through feature classification of disease and iconography transcriptomic datasets. It has been tested in the studies of various diseases, including cancer, schizophrenia, and postpartum hemorrhage^16–20^. Regarding UC, the characteristics of high stability and prediction accuracy of the SVM method have also been proven in previous reports^21, 22^. Another method of ML, the Least Absolute Shrinkage and Selection Operator (LASSO) analysis, is commonly used in the biomarker identification of various carcinoma diseases^23–25^. Previous studies reported that various MLs could be used for the diagnosis prediction of UC, such as PCA analysis^26^ for metabolomics, the GBM analysis^27^ for microbiota, RF analysis^28^ for gene diagnosis, and NN analysis^29^ for immune-related signature. However, no studies identified the useful genes for UC diagnosis prediction based on the comparison among different ML techniques^30^. It is highly recommended to combine or compare different methods to increase the accuracy of classification and prediction for the diagnosis research^31–33^.

Therefore, according to the gene expression omnibus (GEO) database, we aim to select multiple microarrays (including healthy control and UC patients) and to identify the potential useful genes in terms of UC diagnosis through comparing the results from multiple MLs. If applicable, we will further explore the relationship between selected genes and immune cells.

## Methods

### Data collection

Gene Expression Omnibus (GEO, http://www.ncbi.nlm.nih.gov/geo/) is a publicly accessible functional genomics database. We initially searched the keyword of “Ulcerative Colitis”, and then screened the data based on the following criteria: (1) inclusion criteria (i) diagnosed as UC in humans; (ii) derived from colon tissue with transcriptome; (iii) included the healthy control (derived from the UC microarrays with no diseases). (2) exclusion criteria (i) suspected carcinoma or other diseases; (ii) included pharmacological intervention(s) for the treatment of UC patients. The *sva* R package (version 3.36) was used to bias control and to minimize the batch effect among the included various microarrays. After that, we randomly grouped the microarrays as the training set and testing set according to the classic statistical ratio of 6:4. The training data was used to develop the predictive model, while the testing data was used to verify the results of the model.

### Data processing

Based on the training and testing groups, we further analyzed the data. Firstly, we used R package *preprocessCore* (version 1.56.0) for quantile normalization. This process was composed of (1) transferred the primary dataset into a fixed data type of "matrix"; and (2) the function of "normalize. quantiles" was used for quantile normalization. Secondly, we screened the differently expressed genes (DGEs) in both UC patients and healthy subjects of the training set. Then, the functional analysis of DGEs was conducted through Gene Ontology (GO), Kyoto Encyclopedia of Genes and Genomes (KEGG) pathway analysis, Disease Ontology (DO) enrichment analysis, and Gene Set Enrichment Analysis (GSEA). Moreover, six machine-learning algorithms, namely LASSO, SVM, NN, GBM, RF, and PCA, were used to establish the models. Regarding the testing group, we divided two subgroups for verification, including one individualized set and all datasets of the testing group. Finally, a correlation analysis between identified genes and immune cells was performed.

### General statistical consideration

Statistical analysis was conducted with R software (version 4.1.0; https://www.r-project.org/) and the basement of RStudio (version 1.4.1717). For continuous variables, the independent Student’s t-test was adopted if the variables met Gaussian distribution, if not, the *Wilcoxon* test was used. For categorical variables, the *chi-square* test was used, and the *Wilcoxon* test was used for signed-rank variables. The *Pearson* or *Spearman* coefficients were adopted in the correlation analysis. A two-sided *p* value < 0.05 was considered as significant criteria.

### Identify DEGs

The *limma R* package (version 3.44) was used to identify the DEGs. The raw data was processed as log2 transformation after the quantile normalization. The *p* value was adjusted to control the false discovery rate (FDR) based on the method of *Benjamini and Hochberg*. The DEGs were filtered with the criteria of the absolute value of fold change > 1 (|logFC| > 1) and FDR<0.05 (Student’s *t* test).

### Functional enrichment analysis

Functional enrichment analysis was conducted to compare the DEGs between the UC group and the healthy subjects, specifically including Gene Ontology (GO), Kyoto Encyclopedia of Genes and Genomes (KEGG) pathway analysis, Disease Ontology (DO) enrichment analysis, and Gene Set Enrichment Analysis (GSEA) based on *clusterProfiler*, *DOSE*, and *enrichplot* package of R with version 3.16.1, 3.14.0, and 1.8.1 respectively. The GO consist of molecular function (MF), biological process (BP), and cellular components (CC).

### MLs for the development of predictive models

We adopted six machine-learning algorithms (LASSO, SVM, PCA, RF, GBM, and NN) for the identification of significant predictive genes in DEGs. LASSO was performed to screen the candidate genes with binomial deviance and 10-fold cross-validation for the discrimination of UC patients and healthy subjects with *glmnet* (version 4.1) package. To avoid overfitting, the SVM algorithm was also used to adjust the premium genes with *e1071* R packages (version 1.7–6) and the core of “svmRadial”. Specifically, the R pakage *e1071* was applied to the SVM with the function of "rfe" with "sizes" from 2 to 40 with step size of 3, "rfeControl" with "functions" of "caretFuncs" and "cv", and the "methods" was "svmRadial". The SVM code in RStudion was setting as the followed: " rfe (x = data, y = as.numeric (as.factor (group)), sizes = c (seq (2, 40, by = 2)), rfeControl = rfeControl (functions = caretFuncs, method = "cv"), methods = "svmRadial")". The R package *randomForest* (version 4.6–14) was adopted for the RF algorithm with 100 trees. And the PCA algorithm was performed by *psych* package (version 2.2.3). The variable importance in projection (VIP) values of PCA was used to estimate the importance of genes. The *neuralnet* (version 1.44.2) was used for the NN algorithm with 3 hidden. The *h2o* (version 3.36.0.3) was adopted for the GBM algorithm with 100 trees. The overlapping genes that existed in these two algorithm groups were included and the expression levels of candidate genes were further validated in the testing group. Furthermore, the SMOTE with R package *DMwR* (version 0.4.1) was used to expand the sample size when an imbalanced sample size appeared between the two groups. To estimate the prediction value for UC diagnosis, we used the *pROC* package (Version 1.17.0.1) in R. AUC of ROC was calculated to judge the accuracy of the predictive model. The greater value of AUC presents the higher accuracy of the predictive model. Additionally, the error rate was added for the assessment of accuracy among various MLs, and the lowest value of error rate could be indicated a better classification capacity.

### Correlation analysis

To quantify the relative proportions of immune cells from the gene expression profiles, CIBERSORT (https://cibersortx.stanford.edu/), a bioinformatics algorithm, was conducted for correlation analysis. The putative abundance of immune cells was estimated using a reference set with 22 types of immune cell subtypes with 1,000 permutations. Correlation analysis and visualization of these 22 types of immune cells were performed using the *corrplot* R package (version 0.84). Using the *vioplot* R package (version 0.3.5), violin plots visualized the differences of immune cells between the UC group and the healthy control cohort. The *Spearman's* rank correlation analysis in R software was performed. The *ggplot2* R package (version 3.3.5) was adopted to visualize infiltrating associations between various immune cells.

## Results

### Search

**Figure 1:**
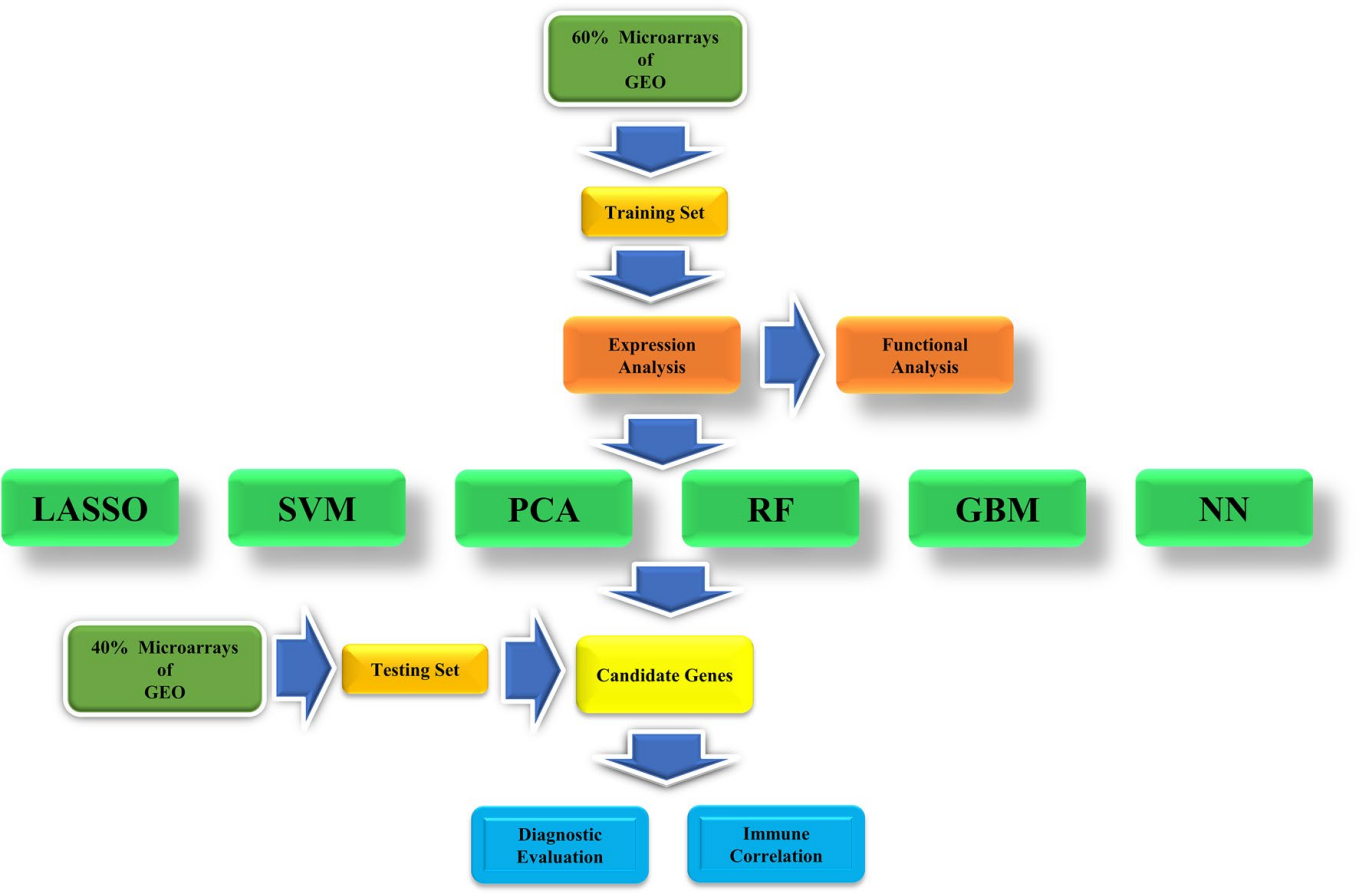
The workflow of the analysis steps.

According to the inclusion and exclusion criteria, a total of ten microarrays (526 individuals), including GSE48634, GSE6731, GSE114527, GSE13367, GSE36807, GSE3629, GSE53306, GSE87473, GSE74265, and GSE96665 were included for subsequent analysis. Based on the random ratio of 6:4, we developed the training set with 6 microarrays (201 UC patients and 106 healthy subjects), including GSE48634, GSE6731, GSE114527, GSE13367, GSE36807, and GSE3629, and further established the predictive model. Meanwhile, we developed the testing set with four microarrays (186 UC patients and 33 healthy subjects), including GSE53306, GSE87473, GSE74265, and GSE96665, and further tested the results of the model. In addition, as the microarray GSE87473 included the largest sample size in the testing set, we also selected it as a separate group for the model testing. With the datasets, we further processed the following analysis just shown in the workflow (Fig. 1).

### Identify DEGs

**Figure 2:**
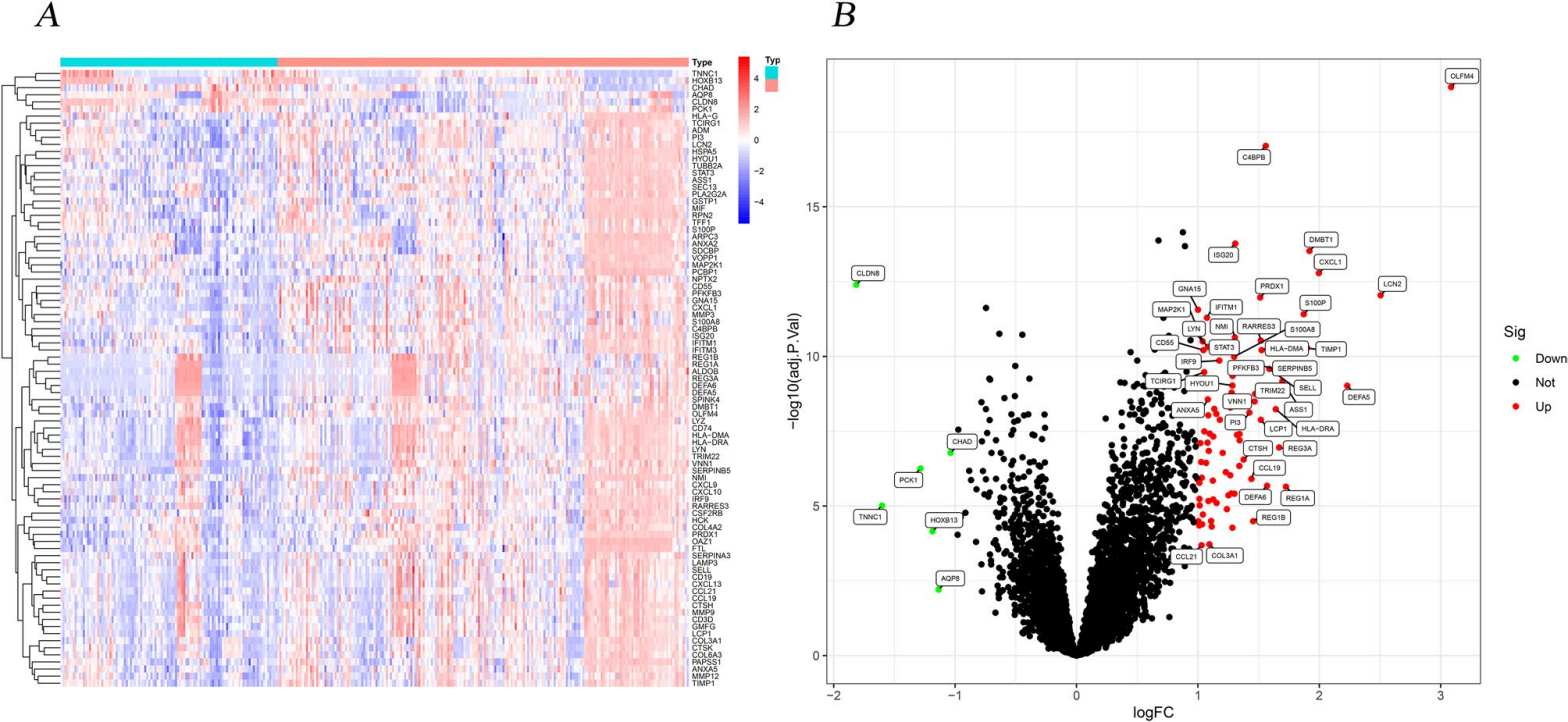
The 87 DEGs distributed in both UC group and healthy group. (**A**. Heatmap; **B**. Volcano diagram.). Note: R software (version 4.1.0; https://www.r-project.org/) was used to create the maps, including R package pheatmap (version 1.0.12; https://cran.r-project.org/web/packages/pheatmap/index.html) for heatmap and ggplot2 (version 3.35; https://cran.r-project.org/web/packages/ggplot2/index.html) for volcano plot, respectively.

Among the training group, we identified a total of 87 DEGs with biological significance. Details are provided in Appendix 1. Compared to the healthy control, there are 81 genes presented as up-regulated and 6 genes shown as down-regulated in the UC patients. Generally, the bigger absolute value of LogFC and adjusted *P* value of Log10 indicates a greater difference between the two groups. Therefore, OLFM4, C4BPB, and CLDN8 distributed in the margin of the heatmap indicated an obvious difference between the two groups (Fig. 2).

### Functional enrichment analysis

**Figure 3:**
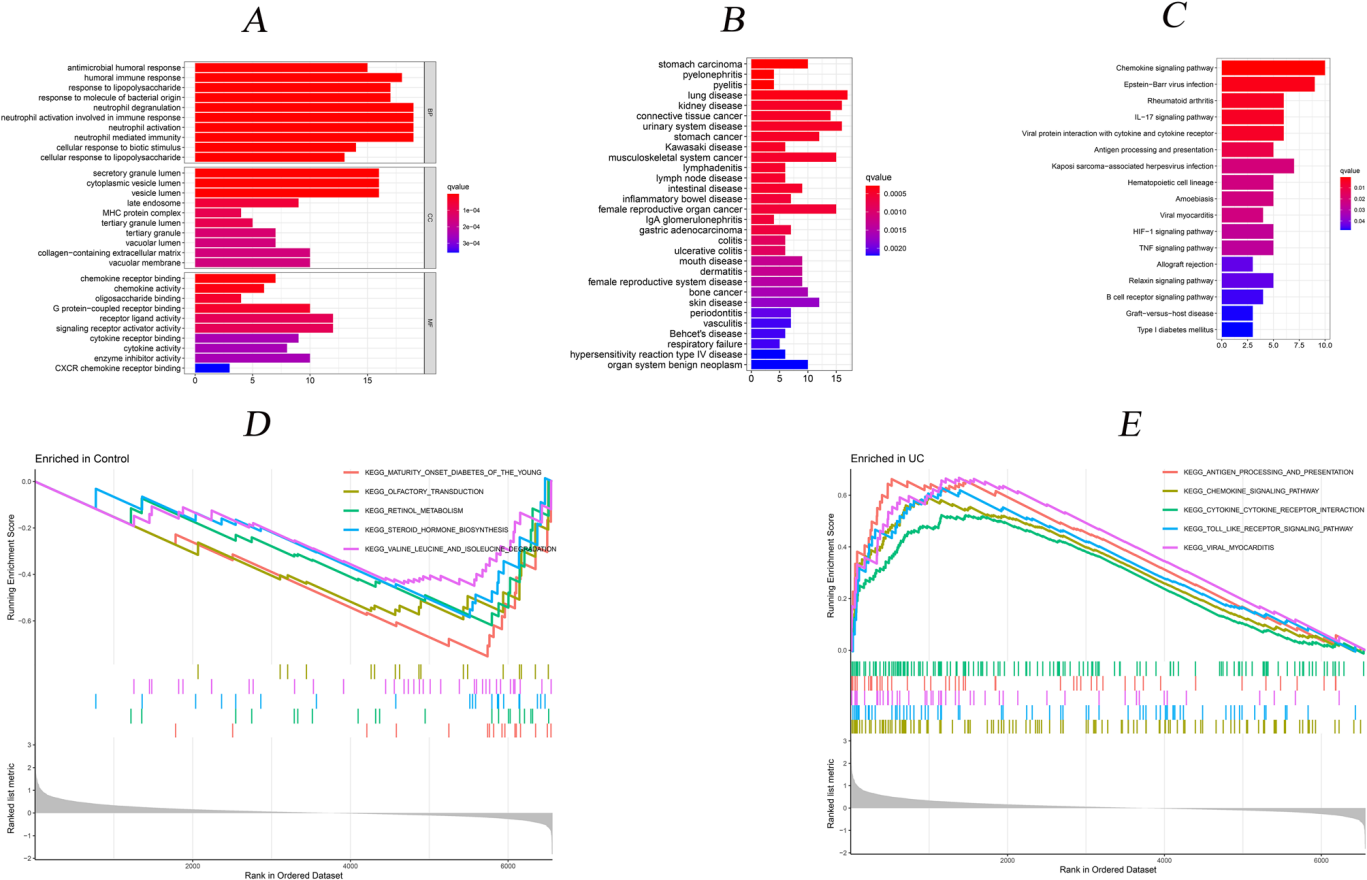
Functional enrichment analysis. (**A**. The top 10 most significantly enriched GO terms; **B**. The top 30 most significantly enriched DO terms; **C**. The 17 significantly enriched KEGG pathways; **D**. The top 5 GSEA-KEGG enrichment in healthy group; **E**. The top 5 GSEA-KEGG enrichment in UC group).

In both UC patients and healthy subjects, we have identified the top 10 GO terms, top 30 DO terms, 17 significant KEGG pathways, and the top 5 single cohorts of GSEA (Fig. 3). Regarding 10 GO terms, the top 3 presented the significant enrichments in the antimicrobial humoral response, humoral immune response, and response to lipopolysaccharide. Regarding 30 DO terms, the top 3 presented the significant enrichments in the stomach carcinoma, pyelonephritis, and pyelitis, respectively. Among KEGG pathways, the top 3 presented the significant enrichments in the Chemokine signaling pathway, Epstein–Barr virus infection, and Rheumatoid arthritis. In terms of GESA among the healthy group, the top 3 also presented the significant enrichments in the Maturity Onset Diabetes Of The Young, Olfactory Transduction, and Retinol Metabolism. Compared to the GESA of the UC group, the top 3 presented significant enrichments in the Antigen Processing And Presentation, Chemokine Signaling Pathway, and Cytokine Cytokine Receptor Interaction.

### Six machine-learning algorithms for candidate genes

**Figure 4:**
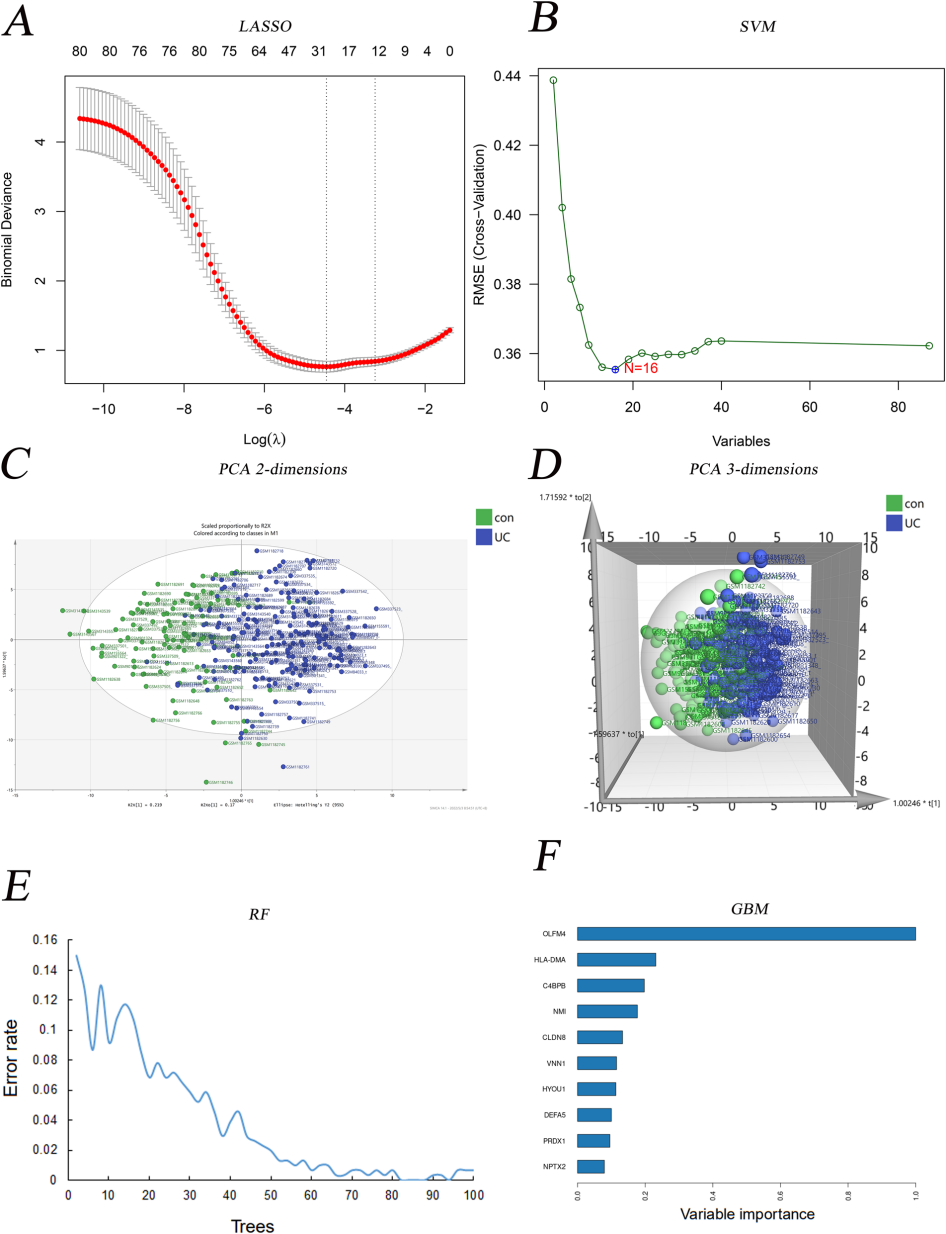
Six MLs for DGEs comparison. (**A**. LASSO for 27 prognostic DGEs; **B**. SVM for 16 prognostic DGEs; **C**. PCA for classification in 2 dimensions; **D**. PCA for classification in 3 dimensions; **E**. The error rate of RF with 100 trees; **F**. The top 10 weighted genes in GBM).

In this study, six predictive models, including LASSO, SVM, PCA, RF, NN, and GBM were successfully established, respectively (Fig. 4). We identified 27 candidate genes through the 10-fold cross-validations of binomial deviance (Fig. 4A) and minimum lambda 0.01162648 of the LASSO algorithm (Appendix 2). In comparison, 16 candidate genes were identified based on the SVM algorithm (Fig. 4B) with *svmRadial* function. Furthermore, the PCA analysis (Fig. 4C-D) indicated that the two groups of UC patients and healthy control were distributed in different quadrants with obvious discrimination among 2 and 3 dimensions (Fig. 4C,D). With the increasing trees of RF analysis, the error rate presented decreased (Fig. 4E). In GBM (Fig. 4F), the various important genes indicated that OlFM4, HLA-DMA, and C4BPB showed a dominant weight proportion.

**Figure 5:**
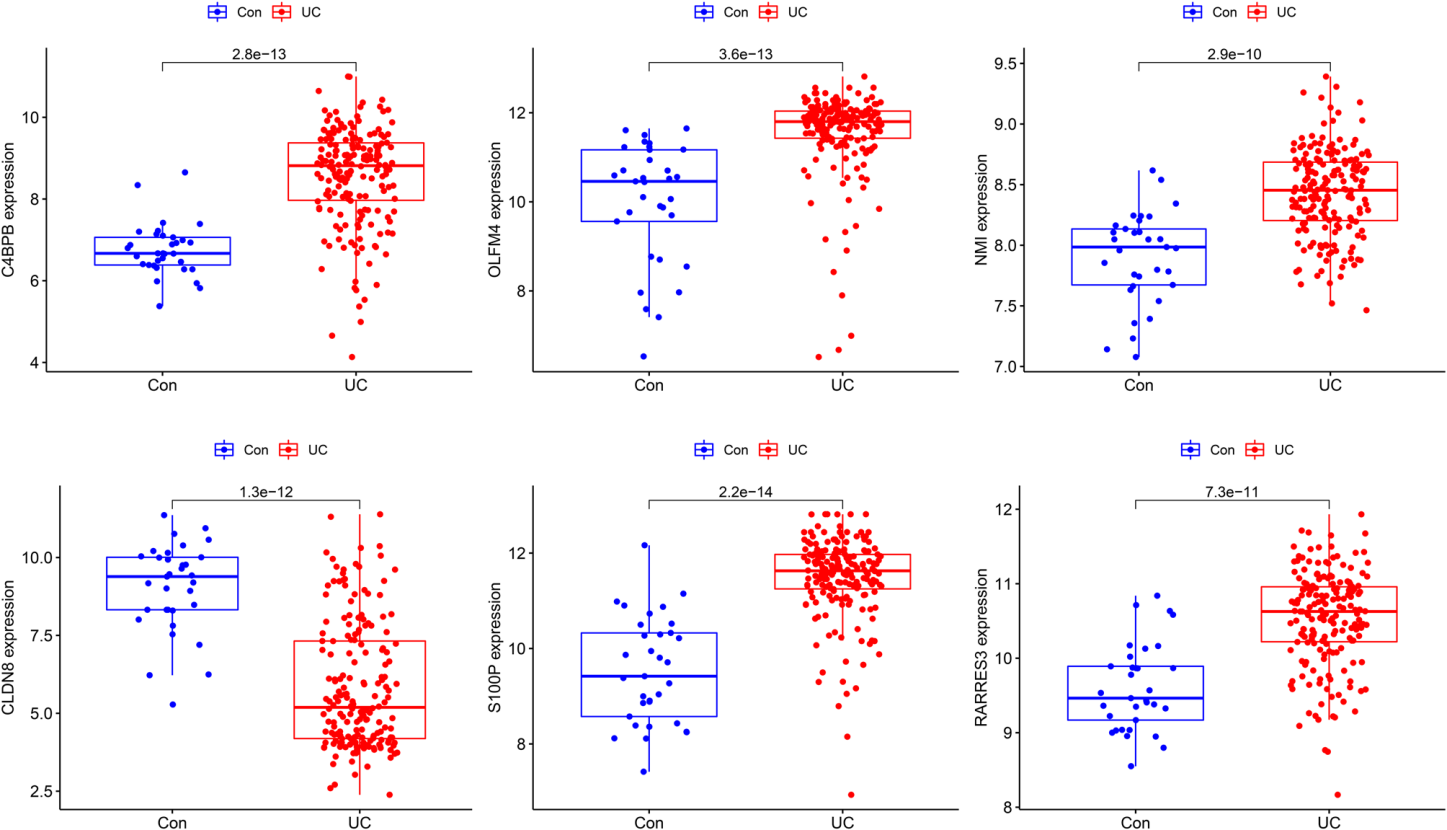
Results comparison of 6 DGEs in testing groups.

As four MLs (SVM, RF, NN, and GBM) were used for classification, we adopted the calculated error rate for model evaluation. The SVM presented the lowest value among the four MLs (Table 1). Moreover, we calculated the top 20 weighted genes in a total of six MLs (Table 2). According to the criteria of counts frequency > 4, there were six genes finally selected, including C4BPB, OLFM4, NMI, CLDN8, S100P, and RARRES3. All of them presented significance in UC group and healthy group (Fig. 5).

**Table 1:** Error rate in different machine learnings.

Machine-learning	Error rate (%)
SVM	**0.16**
RF	0.65
GBM	0.98
NN	0.17

SVM, Support Vector Machine; RF, Random forest; GBM, Gradient boosting machine; NN, Neural network.

Bold value indicates the lowest value.

**Table 2:** The top 20 weighted genes selected from different machine-learnings.

LASSO		PCA		GBM		RF		NN		SVM	
Genes	Weight	Genes	Weight	Genes	Weight	Genes	Weight	Genes	Weight	Genes	Weight
S100P	0.52	C4BPA	1.85	OLFM4	1	OLFM4	3.89	TUBB2A	− 2.39	OLFM4	8.87
RARRES3	0.42	RIPK2	1.85	HLA-DMA	0.23	C4BPB	3.7	TIMP1	2.25	C4BPB	3.37
IFITM3	− 0.31	PYY	1.85	C4BPB	0.2	ISG20	1.63	CCL19	− 2.25	NMI	2.13
CD19	0.29	REG3A	1.85	NMI	0.18	DMBT1	2.43	DEFA6	− 2.01	HLA-DMA	1.96
CHAD	− 0.28	DUSP10	1.85	CLDN8	0.13	CXCL1	1.08	CD55	1.87	VNN1	1.78
NMI	0.24	CNTNAP2	1.84	VNN1	0.12	CLDN8	2.46	CXCL9	1.77	DEFA5	1.78
PLA2G2A	− 0.24	ATP2C2	1.84	HYOU1	0.11	LCN2	0.69	IFITM1	1.7	S100P	1.77
C4BPB	0.19	LRRN2	1.84	DEFA5	0.1	PRDX1	2.67	PCBP1	1.65	PRDX1	1.65
HYOU1	0.19	CHI3L2	1.83	PRDX1	0.1	GNA15	1.01	AQP8	1.64	CLDN8	1.55
VNN1	0.18	TRIM22	1.83	NPTX2	0.08	S100P	2.44	FTL	1.48	REG3A	1.38
NPTX2	0.18	ALOX5	1.83	S100P	0.08	IFITM1	2.03	ASS1	1.4	IRF9	1.34
DMBT1	0.17	OAZ1	1.83	RARRES3	0.08	NMI	3.47	HSPA5	1.34	HYOU1	1.32
OLFM4	0.15	ZNF189	1.82	CXCL1	0.07	RARRES3	1.96	ADM	− 1.34	CXCL1	1.2
CSF2RB	0.15	STAT3	1.82	DEFA6	0.05	MAP2K1	0.93	C4BPB	1.33	NPTX2	1.14
COL6A3	− 0.12	ZNF143	1.82	REG3A	0.05	LYN	1.54	ISG20	1.31	CD55	1.1
PCK1	− 0.11	GPR161	1.82	CHAD	0.05	STAT3	1.35	SDCBP	1.25	RARRES3	0.94
SERPINA3	− 0.08	SWAP70	1.82	VOPP1	0.04	TIMP1	1.23	REG1B	− 1.19	ISG20	0.86
CLDN8	− 0.05	ME1	1.82	CD19	0.04	CD55	1.45	TRIM22	− 1.17	CD19	0.86
COL4A2	0.04	BIRC3	1.82	PCK1	0.04	HLA-DMA	2.11	SERPINA3	1.09	HLA-DRA	0.85
SPINK4	− 0.04	ADRA2A	1.81	HLA-DRA	0.04	S100A8	0.64	CTSK	1.07	SELL	0.81

LASSO, Least Absolute Shrinkage and Selection Operator; PCA, principal component analysis; GBM, Gradient boosting machine; RF, Random forest; NN, Neural network, SVM, Support Vector Machine.

Different MLS process different weights, and negative weights in LASSO and NN that we sort the weighted genes with absolute value.

### Evaluation of the models

**Figure 6:**
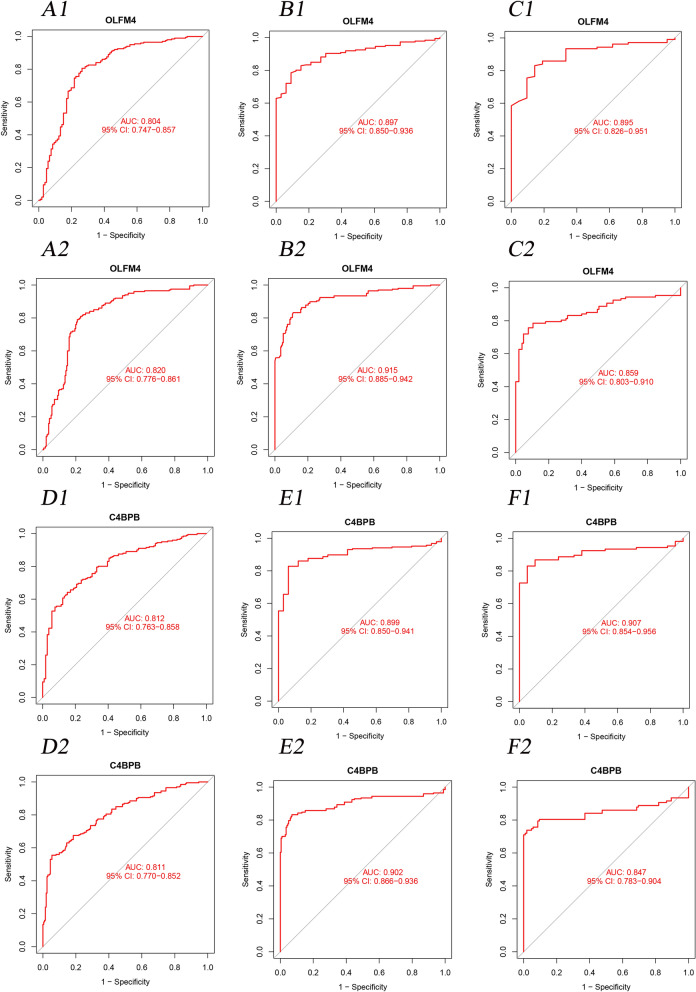
The ROC curve of OLFM4 and C4BPB between two groups. (**A1**. The ROC curve of OLFM4 in training group; **A2**. The ROC curve of OLFM4 in SMOTE-training group; **B1**. The ROC curve of OLFM4 in the testing group; **B2**. The ROC curve of OLFM4 in SMOTE-testing group; **C1**. The ROC curve of OLFM4 in GSE87473 group; **C2**. The ROC curve of OLFM4 in SMOTE-GSE87473 group; **D1**. The ROC curve of C4BPB in training group; **D2**. The ROC curve of C4BPB in SMOTE-training group; **E1**. The ROC curve of C4BPB in the testing group; **E2**. The ROC curve of C4BPB in the SMOTE-testing group; **F1**. The ROC curve of C4BPB in the GSE87473 group; **F2**. The ROC curve of C4BPB in the SMOTE-GSE87473 group).

We adopted the ROC curve and AUC values to assess the diagnosis value of the model. When we set the 6 genes (as mentioned above) into the ROC curve (Appendix 3), the results showed that the AUC values of OLFM4 and C4BPB were higher than 0.8 in the training group, testing group, and individual GSE87473 testing group (Fig. 6A1-F1). In addition, aim to reduce the potential bias from imbalanced sample size in different groups, we selected the SMOTE technique for our analysis. For the training group, we expanded the 1:1 ratio for the UC patients (n=201) and healthy controls (n=201), while in the testing group, we have 197 UC patients vs 198 healthy controls. Regarding the GSE87473, we expanded to 107 patients and 105 healthy controls. As indicated in Fig. 6A2-F2, both the primary datasets and SMOTE datasets showed a good AUC >0.8.

### Correlation analysis

**Figure 7:**
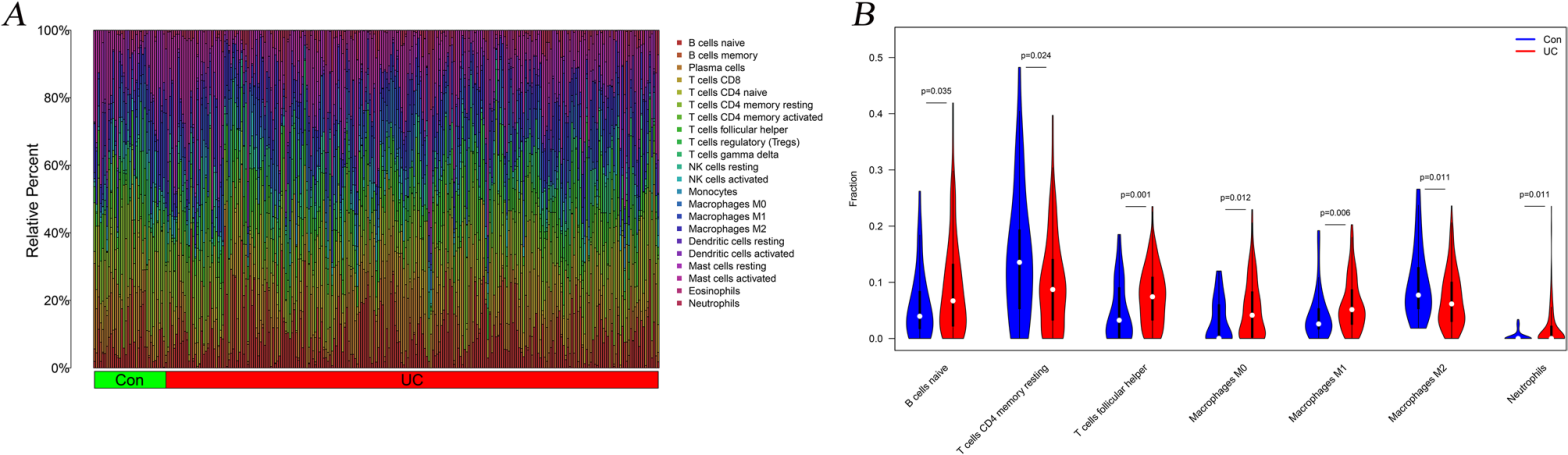
The immune correlation landscape for the ten microarrays. (**A**. Barplot for the 22 immune cells; **B**. Violin plot among two groups in 7 immune cells).

To analyze the relationship between ten microarrays and 22 immune cells, we demonstrated the relative percentage of immune cells among 526 samples of ten microarrays (Fig. 7A). Then, the significant immune proportions of the UC and healthy groups indicated that there were 7 types of immune cells, including B cells naive, T cells CD4 memory resting, T cells follicular helper, Macrophages M0, Macrophages M1, and Macrophages M2, Neutrophils (Fig. 7B).

**Figure 8:**
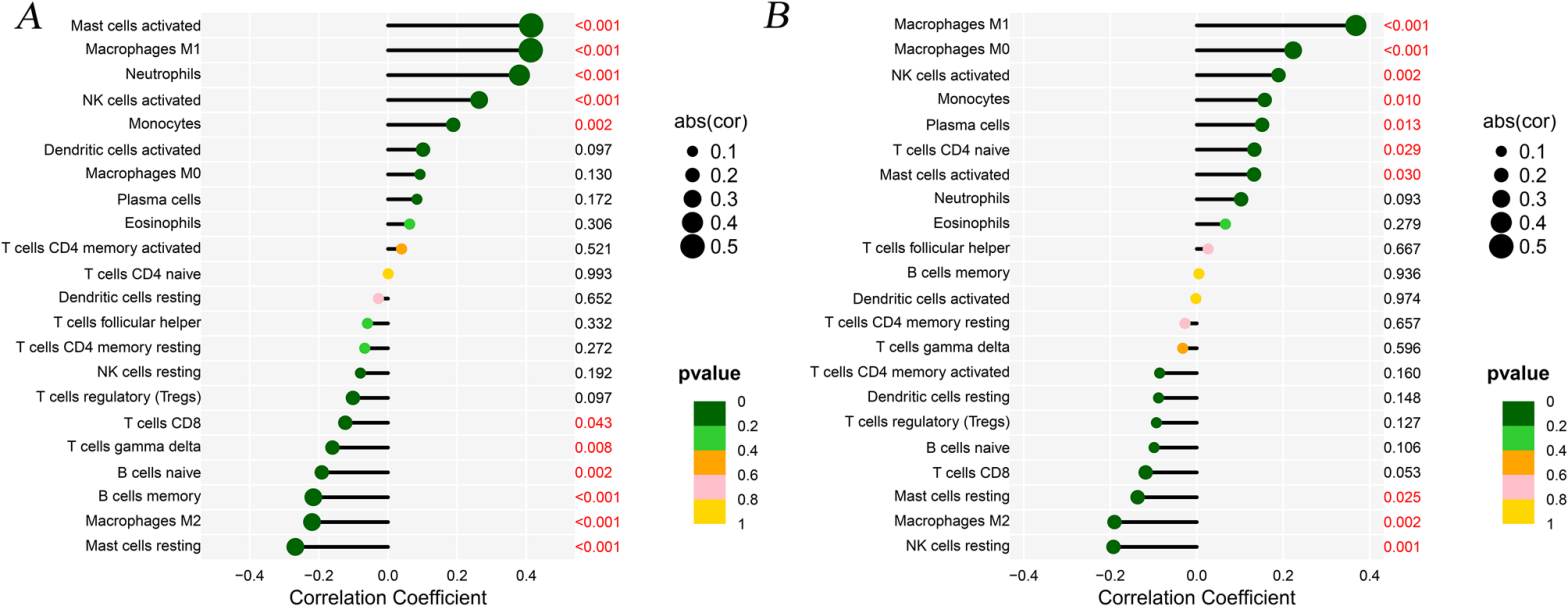
The lollipop figure in the immune correlation of C4BPB and OLFM4. (**A**. The immune correlation in C4BPB; **B**. The immune correlation in OLFM4).

Moreover, we analyzed the correlations between 22 immunize cells and the two important genes of OLFM4 and C4BPB through the *Spearman* analysis (Appendix 4). The significant results were presented in the following 6 types of immune cells, including Macrophages M1, Macrophages M2, Mast cells activated, Mast cells resting, Monocytes, and NK cells activated (Fig. 8).

## Discussion

In this study, we included ten microarrays with 526 samples for data analysis, and further selected a total of 87 DEGs. Based on the six MLs, we successfully established the predictive models and identified two useful genes in the diagnosis of UC, namely OLFM4 and C4BPB. The AUC values of C4BPB (average 0.873) were 0.812, 0.899, and 0.907 in the training group, testing group, and individual GSE87473 testing group, respectively. Compared to the previous studies, C4BPB presented the diagnosis value in Crohn's disease^34^. But this study extended its scope to UC. Additionally, the AUC values of OLFM4 (average 0.865) were 0.804, 0.897, and 0.895 in the training group, testing group, and individual GSE87473 testing group, respectively. In previous studies, OLFM4, as a cancer stemness gene induced by IL-22, was highly distributed in primary sclerosing cholangitis-associated ulcerative colitis^35^. It was also overexpressed in the active IBD^36^. In this study, we added a new result in terms of diagnosis values of OLFM4 for UC patients.

Regarding the correlations analysis, these two genes (e.g., OLFM4 and C4BPB) presented significant associations with 6 types of immune cells, including Macrophages M1, Macrophages M2, Mast cells activated, Mast cells resting, Monocytes, and NK cells activated. Some scholars have found that the UC patients presented an increasing percentage of CD23 B naive cells, compared to the normal individuals^5^. Regulatory T cells were also a key factor that exacerbated UC through immune imbalance^37^. Compared to Crohn's disease, the colonic mucosa samples in UC patients showed the expansion of IL17A+ CD161+ effector memory T cells and IL17A+ T-regulatory cells, expansion of HLA-DR+CD56+ granulocytes, and reductions in type 3 innate lymphoid cells^38^. The regulation of T cells for UC patients based on Bcl-6 and IL-21 could help to avoid the occurrence and development of IBD^39^. In a previous survey that included a global immune cell landscape of UC patients’ tissue, the results identified the increasing number of neutrophils, T CD4 memory-activated cells, active dendritic cells, and M0 macrophages, and decreasing number of T CD8, Tregs, B memory, and M2 macrophages^40^.

Through the functional enrichment analysis, we found three pathways, including Chemokine signaling pathway, Epstein–Barr virus infection pathways, and Rheumatoid arthritis pathways, might be closely related to the progress of UC. The previous study had emphasized the potential role of the Chemokine signaling pathway in the up-regulation of UC patients and further proposed the CXCL8-CXCR137 (a type of chemokine) in the treatment of UC^41^. Although Epstein–Barr virus infection might trigger several immune dysfunctions, such as natural killer/T cell lymphoma arising, hemophagocytic lymphohistiocytosis, and malignancies, few studies focused on UC previously^41–43^. Furthermore, Epstein Barr Virus might be useful in the development of vaccines and immune cell therapy for EBV-Associated diseases, especially for several immune-related diseases^44^. The prognosis of Epstein–Barr virus infection in UC was less paid attention to^45^. In this study, the KEGG pathways results indicated that more studies of the Epstein–Barr virus infection pathway in UC could be conducted^46^. Particularly, IBD patients have a higher risk to develop autoimmune and inflammatory diseases, such as rheumatoid arthritis^47^.

Actually, there are many limitations to using machine learning in a clinical setting. As MLs include multiple factors, especially in statistics, clinical practice, and bioinformatics. To improve the study design and to facilitate the explanation of results from ML analysis, it is recommended to include a variety of experts of authors/researchers in a study. Individuals with rich clinical experience and MLs technique background are also conducive to playing an important role in clinical MLs studies. In this study, there are some limitations. Firstly, insufficient verification is a common type of limitation in bioinformatics studies. Although we designed testing groups to assess the stability of the predictive model based on AUC values, and included ten microarrays to increase the sample size in this study, more research works, either in clinical trials or animal experiments, should be conducted to obtain solid verifications for these predictive results. Secondly, the machine-learning model itself includes some limitations, such as the black box phenomenon^48^, particularly in the NN method which includes many layers, such as an input layer, an output layer, and hidden layers (count fluctuating)^49, 50^. Among them, the characteristics of the hidden layers are hard to identify^51^. Thirdly, we have limited information about the clinical features, such as the patient's age^30^, ethnicity and race^52, 53^ and stage of UC. Generally, some detailed information impacts the algorithm bias. Thus, further subgroup analysis could be included to identify more useful results in future research.

## Conclusion

In this study, we found two useful genes of OLFM4 and C4BPB which may help to identify UC patients. Further verification studies could be conducted.

## Supplementary Information

Supplementary Information.

## Data Availability

The datasets generated and analysed during the current study are available in the Gene Expression Omnibus (GEO) (https://www.ncbi.nlm.nih.gov/geo/), and all the multiple micorarrays of GSE48634, GSE6731, GSE114527, GSE13367, GSE36807, GSE3629, GSE53306, GSE87473, GSE74265, and GSE96665 were derived from this database.

## Abbreviations

UC: Ulcerative colitis
GEO: Gene expression omnibus
IBD: Inflammatory bowel disease
ML: Machine learning
LASSO: The least absolute shrinkage and selection operator
SVM: Support vector machine
RF: Random forest
GBM: Gradient boosting machine
NN: Neural network
PCA: Principal component analysis
DEGs: Differential expression genes
GO: Gene ontology
KEGG: Kyoto encyclopedia of genes and genomes
DO: Disease ontology
GSEA: Gene set enrichment analysis
MF: Molecular function
BP: Biological process
CC: Cellular components
ROC: Receiver operating characteristic
AUC: Area under curve

## References

[1] Kornbluth, A. & Sachar, D. B. Ulcerative colitis practice guidelines in adults: American College Of Gastroenterology, Practice Parameters Committee. *Am. J. Gastroenterol.* **105**, 501–523; quiz 524. 10.1038/ajg.2009.727 (2010). 10.1038/ajg.2009.727 20068560

[2] Harbord M (2017). Corrigendum: Third European evidence-based consensus on diagnosis and management of ulcerative colitis. Part 2: Current management. J. Crohns Colitis..

[3] Tian M, Ma P, Zhang Y, Mi Y, Fan D (2020). Ginsenoside Rk3 alleviated DSS-induced ulcerative colitis by protecting colon barrier and inhibiting NLRP3 inflammasome pathway. Int. Immunopharmacol..

[4] Ma C (2022). Systematic review: Disease activity indices for immune checkpoint inhibitor-associated enterocolitis. Aliment. Pharmacol. Ther..

[5] Rabe H (2019). Distinct patterns of naive, activated and memory T and B cells in blood of patients with ulcerative colitis or Crohn's disease. Clin. Exp. Immunol..

[6] Fu Y (2021). Intestinal CD11b(+) B cells ameliorate colitis by secreting immunoglobulin A. Front. Immunol..

[7] Choi CH (2017). Second Korean guidelines for the management of ulcerative colitis. Intest. Res..

[8] Peyrin-Biroulet L (2022). Etrolizumab as induction and maintenance therapy for ulcerative colitis in patients previously treated with tumour necrosis factor inhibitors (HICKORY): A phase 3, randomised, controlled trial. Lancet Gastroenterol. Hepatol..

[9] Ko CW (2019). AGA clinical practice guidelines on the management of mild-to-moderate ulcerative colitis. Gastroenterology.

[10] Lai L, Li H, Feng Q, Shen J, Ran Z (2021). Multi-factor mediated functional modules identify novel classification of ulcerative colitis and functional gene panel. Sci. Rep..

[11] Zhang D, Yan P, Han T, Cheng X, Li J (2021). Identification of key genes and biological processes contributing to colitis associated dysplasia in ulcerative colitis. PeerJ.

[12] Kim SH (2021). Effectiveness and drug retention of biologic disease modifying antirheumatic drugs in Korean patients with late onset ankylosing spondylitis. Sci. Rep..

[13] Lu J (2022). Identification of diagnostic signatures in ulcerative colitis patients via bioinformatic analysis integrated with machine learning. Hum. Cell..

[14] Su S, Kong W, Zhang W, Wang W, Guo H (2020). Integrated analysis of DNA methylation and gene expression profiles identified S100A9 as a potential biomarker in ulcerative colitis. Biosci. Rep.

[15] Gubatan J (2021). Artificial intelligence applications in inflammatory bowel disease: Emerging technologies and future directions. World J. Gastroenterol..

[16] Kraszewski S, Szczurek W, Szymczak J, Reguła M, Neubauer K (2021). Machine learning prediction model for inflammatory bowel disease based on laboratory markers working. Model in a Discovery Cohort Study. J. Clin. Med..

[17] Akazawa M, Hashimoto K, Katsuhiko N, Kaname Y (2021). Machine learning approach for the prediction of postpartum hemorrhage in vaginal birth. Sci. Rep..

[18] Cruz-Martinez C, Reyes-Garcia CA, Vanello N (2022). A novel event-related fMRI supervoxels-based representation and its application to schizophrenia diagnosis. Comput. Methods Programs Biomed..

[19] Stryker S, Kapadia AJ, Greenberg JA (2022). Application of machine learning classifiers to X-ray diffraction imaging with medically relevant phantoms. Med0 Phys..

[20] Xv Y (2021). Machine learning-based CT radiomics approach for predicting WHO/ISUP nuclear grade of clear cell renal cell carcinoma: An exploratory and comparative study. Insights Imaging.

[21] Al-Harazi O, Kaya IH, El Allali A, Colak D (2021). A network-based methodology to identify subnetwork markers for diagnosis and prognosis of colorectal cancer. Front. Genet..

[22] Khorasani HM, Usefi H, Peña-Castillo L (2020). Detecting ulcerative colitis from colon samples using efficient feature selection and machine learning. Sci. Rep..

[23] Ding H (2017). In vivo analysis of mucosal lipids reveals histological disease activity in ulcerative colitis using endoscope-coupled Raman spectroscopy. Biomed. Opt. Express.

[24] Fujii T, Maehara K, Fujita M, Ohkawa Y (2021). Discriminative feature of cells characterizes cell populations of interest by a small subset of genes. PLoS Comput. Biol..

[25] Jun H, ZeXin Z (2021). Screening of pyroptosis-related genes influencing the therapeutic effect of dehydroabietic acid in liver cancer and construction of a survival nomogram. Biochem. Biophys. Res. Commun..

[26] Williams HR (2009). Characterization of inflammatory bowel disease with urinary metabolic profiling. Am. J. Gastroenterol..

[27] Bakir-Gungor B (2022). Inflammatory bowel disease biomarkers of human gut microbiota selected via different feature selection methods. PeerJ.

[28] Olsen J (2009). Diagnosis of ulcerative colitis before onset of inflammation by multivariate modeling of genome-wide gene expression data. Inflamm. Bowel Dis..

[29] Chen X (2021). Artificial neural network analysis-based immune-related signatures of primary non-response to infliximab in patients with ulcerative colitis. Front. Immunol..

[30] Kalkan IH, Dağli U, Oztaş E, Tunç B, Ulker A (2013). Comparison of demographic and clinical characteristics of patients with early vs. adult vs. late onset ulcerative colitis. Eur. J. Intern. Med..

[31] Zhuge L (2022). A novel model based on liquid–liquid phase separation—related genes correlates immune microenvironment profiles and predicts prognosis of lung squamous cell carcinoma. J Clin Lab Anal..

[32] Chen X (2022). MRI-based radiomics model for distinguishing endometrial carcinoma from benign mimics: A multicenter study. Eur J Radiol..

[33] Yu YX (2021). Value of the application of enhanced CT radiomics and machine learning in preoperative prediction of microvascular invasion in hepatocellular carcinoma. Zhonghua Yi Xue Za Zhi.

[34] Dobre M (2018). Differential intestinal mucosa transcriptomic biomarkers for Crohn's disease and ulcerative colitis. J. Immunol. Res..

[35] Neyazi M (2021). Overexpression of cancer-associated stem cell gene OLFM4 in the colonic epithelium of patients with primary sclerosing cholangitis. Inflamm. Bowel Dis..

[36] Gersemann M (2012). Olfactomedin-4 is a glycoprotein secreted into mucus in active IBD. J. Crohns Colitis..

[37] Hanai H (2013). A new paradigm in ulcerative colitis: Regulatory T cells are key factor which induces/exacerbates UC through an immune imbalance. Mol. Immunol..

[38] Mitsialis V (2020). Single-cell analyses of colon and blood reveal distinct immune cell signatures of ulcerative colitis and Crohn's disease. Gastroenterology.

[39] Yang Y (2020). Case report: IL-21 and Bcl-6 regulate the proliferation and secretion of Tfh and Tfr cells in the intestinal germinal center of patients with inflammatory bowel disease. Front Pharmacol..

[40] Penrose HM (2021). Ulcerative colitis immune cell landscapes and differentially expressed gene signatures determine novel regulators and predict clinical response to biologic therapy. Sci. Rep..

[41] Linggi B (2021). Meta-analysis of gene expression disease signatures in colonic biopsy tissue from patients with ulcerative colitis. Sci. Rep..

[42] Zhu Y (2021). CXCL8 chemokine in ulcerative colitis. Biomed. Pharmacother..

[43] Santos AT, Tong J, Steinberg A, Shemen L (2021). Epstein–Barr virus-induced natural killer/T cell lymphoma arising in tonsil and cervical node tissue. BMJ Case Rep..

[44] Xu L, Guo X, Guan H (2022). Serious consequences of Epstein–Barr virus infection: Hemophagocytic lymphohistocytosis. Int. J. Lab Hematol..

[45] Bauer M, Jasinski-Bergner S, Mandelboim O, Wickenhauser C, Seliger B (2021). Epstein–Barr virus-associated malignancies and immune escape: The role of the tumor microenvironment and tumor cell evasion strategies. Cancers (Basel).

[46] Cui X, Snapper CM (2021). Epstein Barr virus: Development of vaccines and immune cell therapy for EBV-associated diseases. Front Immunol..

[47] Liu Y (2021). Clinical features of intestinal ulcers complicated by Epstein–Barr virus infection: Importance of active infection. Dis Markers.

[48] Regazzoni F, Chapelle D, Moireau P (2021). Combining data assimilation and machine learning to build data-driven models for unknown long time dynamics—applications in cardiovascular modeling. Int. J. Numer. Method Biomed. Eng..

[49] Peng JC, Ran ZH, Shen J (2015). Seasonal variation in onset and relapse of IBD and a model to predict the frequency of onset, relapse, and severity of IBD based on artificial neural network. Int. J. Colorectal Dis..

[50] Kang T, Ding W, Zhang L, Ziemek D, Zarringhalam K (2017). A biological network-based regularized artificial neural network model for robust phenotype prediction from gene expression data. BMC Bioinform..

[51] Ozawa T (2019). Novel computer-assisted diagnosis system for endoscopic disease activity in patients with ulcerative colitis. Gastrointest. Endosc..

[52] Jiang L (2007). Risk factors for ulcerative colitis in a Chinese population: An age-matched and sex-matched case-control study. J. Clin. Gastroenterol..

[53] Yamamoto-Furusho JK (2011). Interleukin 1 β (IL-1B) and IL-1 antagonist receptor (IL-1RN) gene polymorphisms are associated with the genetic susceptibility and steroid dependence in patients with ulcerative colitis. J. Clin. Gastroenterol..

