# Supplementary material for: Identification of useful genes from multiple microarrays for ulcerative colitis diagnosis based on machine learning methods
Source: Sci Rep. 2022 Jun 15;12:9962. doi: 10.1038/s41598-022-14048-6 (PMC9200771; doi:10.1038/s41598-022-14048-6)
Supplement: Supplementary file 1 — Supplementary Information. [file 41598_2022_14048_MOESM1_ESM.pdf]

OLFM4 and C4BPB, the Novel Diagnosis Gene Signatures and Immune Correlation for Ulcerative Colitis with Integrated Analysis of Multiple Microarray Studies

Content

Table.1 The 87 differently expressed genes in healthy control and UC cohort.

Table.2 The subsequently screened characteristic with LASSO regression.

Fig.1 The six DGEs into ROC curve.

A. The ROC curve of six DGEs in training group.

B. The ROC curve of six DGEs in testing group.

C. The ROC curve of six DGEs in GSE87473 group.

Table.3 The immune correlation in OLFM4 and C4BPB.

Table.1 The 87 differently expressed genes in healthy control and UC cohort.

ID	logFC	AveExpr	t	P.Value	adj.P.Val	B
CLDN8	-1.814768497	8.220200709	-8.55781	5.52E-16	4.02E-13	25.69652
TNNC1	-1.601797172	6.62836574	-5.09171	6.19E-07	9.74E-06	5.648968
PCK1	-1.285062472	8.185118102	-5.76299	2.00E-08	5.62E-07	8.918545
HOXB13	-1.185806867	8.159745724	-4.57725	6.84E-06	7.00E-05	3.374855
AQP8	-1.135774697	9.339402519	-3.19403	0.001548	0.006196	-1.66141
CHAD	-1.037824548	6.461571829	-6.04223	4.38E-09	1.67E-07	10.37445
GNA15	1.000727035	6.661748827	8.22617	5.48E-15	2.77E-12	23.47382
RPN2	1.002700691	9.482332064	4.786774	2.63E-06	3.24E-05	4.275977
ALDOB	1.011314262	7.655774452	4.701041	3.91E-06	4.47E-05	3.903048
SPINK4	1.011902538	10.90435706	5.505221	7.77E-08	1.68E-06	7.62382
ARPC3	1.012468505	10.74963032	5.212645	3.42E-07	5.78E-06	6.213246
CD3D	1.014711335	8.053071243	5.096058	6.06E-07	9.58E-06	5.669046
CXCL9	1.019692359	7.570292862	6.192167	1.89E-09	7.91E-08	11.17846
OAZ1	1.027144405	12.19068367	5.88228	1.05E-08	3.40E-07	9.533823
CCL21	1.029961601	7.449472391	4.277689	2.52E-05	0.000206	2.147826
VOPP1	1.031978432	8.260436521	5.600779	4.73E-08	1.14E-06	8.098196
TFF1	1.035156178	10.57514032	4.723279	3.53E-06	4.11E-05	3.999222
MAP2K1	1.038879172	8.649711809	7.778124	1.12E-13	3.15E-11	20.56039
MMP3	1.041428416	8.017028248	4.921956	1.40E-06	1.93E-05	4.875733
CD55	1.045736346	8.761643023	7.636885	2.82E-13	6.20E-11	19.66433
TCIRG1	1.051823225	7.982610792	7.32833	2.06E-12	3.38E-10	17.74577
CSF2RB	1.052976854	7.029487595	6.387306	6.21E-10	3.21E-08	12.24762
GMFG	1.067327333	7.601801293	5.863688	1.17E-08	3.64E-07	9.437269
IFITM1	1.074815632	10.48058717	8.105434	1.25E-14	5.12E-12	22.67838
PCBP1	1.079459801	10.31027058	6.198967	1.82E-09	7.66E-08	11.21529
ANXA5	1.082070166	9.734071005	6.904267	2.89E-11	2.75E-09	15.20004
LYN	1.082972435	8.701342852	7.70216	1.84E-13	4.83E-11	20.07709
HCK	1.084836743	7.323210948	6.637545	1.44E-10	9.33E-09	13.65541
HSPA5	1.086669524	9.282858122	5.176356	4.09E-07	6.83E-06	6.042751
COL4A2	1.089786655	8.026876704	6.071264	3.73E-09	1.45E-07	10.52894
COL3A1	1.094895652	9.438348095	4.299852	2.30E-05	0.000191	2.236103
LAMP3	1.097159111	6.852129348	6.34908	7.74E-10	3.82E-08	12.03618
HLA-G	1.108854205	9.293610777	4.793707	2.55E-06	3.18E-05	4.306389
STAT3	1.111832516	8.65057599	7.692181	1.96E-13	4.96E-11	20.01384
FTL	1.113572943	12.40624686	4.679694	4.31E-06	4.86E-05	3.811096
CTSK	1.123122247	7.839003642	5.543714	6.37E-08	1.42E-06	7.814109
GSTP1	1.126329017	10.28402016	5.201455	3.61E-07	6.06E-06	6.160567
CD19	1.126675141	7.20452634	6.299212	1.03E-09	4.76E-08	11.7618
NPTX2	1.13513836	6.575358896	6.748797	7.39E-11	5.71E-09	14.29424
IFITM3	1.152194791	10.76331958	6.669444	1.19E-10	8.38E-09	13.83777

CXCL13	1.153861954	7.887590928	5.140589	4.87E-07	7.90E-06	5.875687
IRF9	1.176491624	9.33510881	7.497966	6.95E-13	1.38E-10	18.79384
SEC13	1.180864127	9.235349734	6.57321	2.10E-10	1.31E-08	13.28959
PAPSS1	1.204439642	7.989200005	6.036788	4.51E-09	1.68E-07	10.34555
ANXA2	1.23014694	10.10964059	5.700935	2.79E-08	7.41E-07	8.602494
MIF	1.238902874	10.22352147	5.024269	8.58E-07	1.28E-05	5.339105
COL6A3	1.252231124	8.770402005	5.280113	2.44E-07	4.39E-06	6.532865
ADM	1.266560348	9.684623155	5.645446	3.74E-08	9.41E-07	8.322206
MMP12	1.26751927	8.773630868	6.776952	6.24E-11	5.12E-09	14.45716
LYZ	1.276665665	9.232820912	5.312867	2.07E-07	3.82E-06	6.689263
VNN1	1.277741726	6.52488932	7.006761	1.54E-11	1.63E-09	15.80539
HYOU1	1.284440909	8.44901357	7.12067	7.60E-12	9.41E-10	16.48566
PLA2G2A	1.284590051	10.33489923	4.654631	4.83E-06	5.33E-05	3.7036
PFKFB3	1.285977899	7.9545831	7.267632	3.02E-12	4.51E-10	17.37482
S100A8	1.298480248	7.545801111	7.548559	5.01E-13	1.03E-10	19.1096
SDCBP	1.301386749	9.802075313	5.309054	2.11E-07	3.88E-06	6.671012
NMI	1.304972105	8.181971426	7.846593	7.09E-14	2.33E-11	20.99871
ISG20	1.307784443	9.783750948	9.083638	1.30E-17	1.70E-14	29.32802
CXCL10	1.322263449	7.072433668	6.326682	8.81E-10	4.19E-08	11.91274
CD74	1.341588605	9.539177066	5.811022	1.55E-08	4.60E-07	9.165085
TUBB2A	1.343192583	8.615597287	6.343139	8.01E-10	3.92E-08	12.00341
SERPINA3	1.343794502	7.401127103	6.236498	1.47E-09	6.40E-08	11.41911
CTSH	1.376774135	8.732174892	5.924891	8.35E-09	2.81E-07	9.756027
PI3	1.424114283	10.44447154	6.691909	1.04E-10	7.56E-09	13.9666
CCL19	1.441433819	7.745895307	5.579002	5.30E-08	1.25E-06	7.989503
REG1B	1.455234475	8.132124445	4.789947	2.60E-06	3.22E-05	4.289891
MMP9	1.467399097	7.890670263	6.869073	3.58E-11	3.21E-09	14.99368
TRIM22	1.47097504	7.694248481	6.987681	1.73E-11	1.80E-09	15.69221
SERPINB5	1.51131716	7.069203098	7.427166	1.10E-12	2.00E-10	18.3544
PRDX1	1.513252131	11.18213003	8.388028	1.80E-15	1.07E-12	24.5518
RARRES3	1.518654286	9.412875451	7.794731	1.00E-13	2.98E-11	20.66647
LCP1	1.519202946	8.930594713	6.570298	2.14E-10	1.32E-08	13.2731
HLA-DMA	1.525328238	9.493362829	7.636194	2.83E-13	6.20E-11	19.65998
C4BPB	1.55929331	7.319900669	10.21055	2.83E-21	9.27E-18	37.50545
DEFA6	1.569836163	7.781988133	5.454736	1.01E-07	2.12E-06	7.375894
SELL	1.58783643	6.800406791	7.376068	1.52E-12	2.62E-10	18.03904
HLA-DRA	1.641893058	11.20430774	6.741682	7.72E-11	5.82E-09	14.25315
REG3A	1.669729025	7.506283338	6.123288	2.79E-09	1.11E-07	10.80721
ASS1	1.695967228	10.13474749	7.187145	5.02E-12	6.72E-10	16.88626
REG1A	1.725579356	9.119742571	5.436587	1.11E-07	2.28E-06	7.287223
S100P	1.87180659	10.92513489	8.165707	8.29E-15	3.88E-12	23.07454
TIMP1	1.900681687	10.03446095	7.676968	2.17E-13	5.28E-11	19.91751
DMBT1	1.919044988	9.262351259	8.959136	3.19E-17	2.99E-14	28.45676
CXCL1	1.996764192	7.433971676	8.701092	2.01E-16	1.65E-13	26.67334

DEFA5	2.230302233	8.436364864	7.111482	8.05E-12	9.61E-10	16.4305
LCN2	2.504959386	10.22767765	8.425917	1.38E-15	9.08E-13	24.80603
OLFM4	3.086237788	10.42001706	10.8783	1.53E-23	1.00E-19	42.56959

Table.2 The subsequently screened characteristic with LASSO regression.

Method	Genes	Condition	Coef
LASSO	OLFM4	lambda.min	0.14751895
LASSO	C4BPB	lambda.min	0.185487713
LASSO	DMBT1	lambda.min	0.173906139
LASSO	CLDN8	lambda.min	-0.053189673
LASSO	PRDX1	lambda.min	0.021082433
LASSO	S100P	lambda.min	0.524508913
LASSO	NMI	lambda.min	0.236032555
LASSO	RARRES3	lambda.min	0.424438671
LASSO	HLA-DMA	lambda.min	0.009897685
LASSO	HYOU1	lambda.min	0.187376225
LASSO	VNN1	lambda.min	0.176034637
LASSO	NPTX2	lambda.min	0.175898179
LASSO	IFITM3	lambda.min	-0.313196821
LASSO	CSF2RB	lambda.min	0.152102962
LASSO	CD19	lambda.min	0.291291482
LASSO	SERPINA3	lambda.min	-0.075735023
LASSO	COL4A2	lambda.min	0.036256726
LASSO	CHAD	lambda.min	-0.277883251
LASSO	OAZ1	lambda.min	0.017195566
LASSO	PCK1	lambda.min	-0.111599485
LASSO	CCL19	lambda.min	0.024118438
LASSO	SPINK4	lambda.min	-0.042505499
LASSO	COL6A3	lambda.min	-0.124202129
LASSO	HLA-G	lambda.min	0.03043852
LASSO	FTL	lambda.min	0.024739742
LASSO	PLA2G2A	lambda.min	-0.236351021
LASSO	CCL21	lambda.min	0.017557336

Fig.1 The six DGEs into ROC curve

A. The ROC curve of six DGEs in training group.

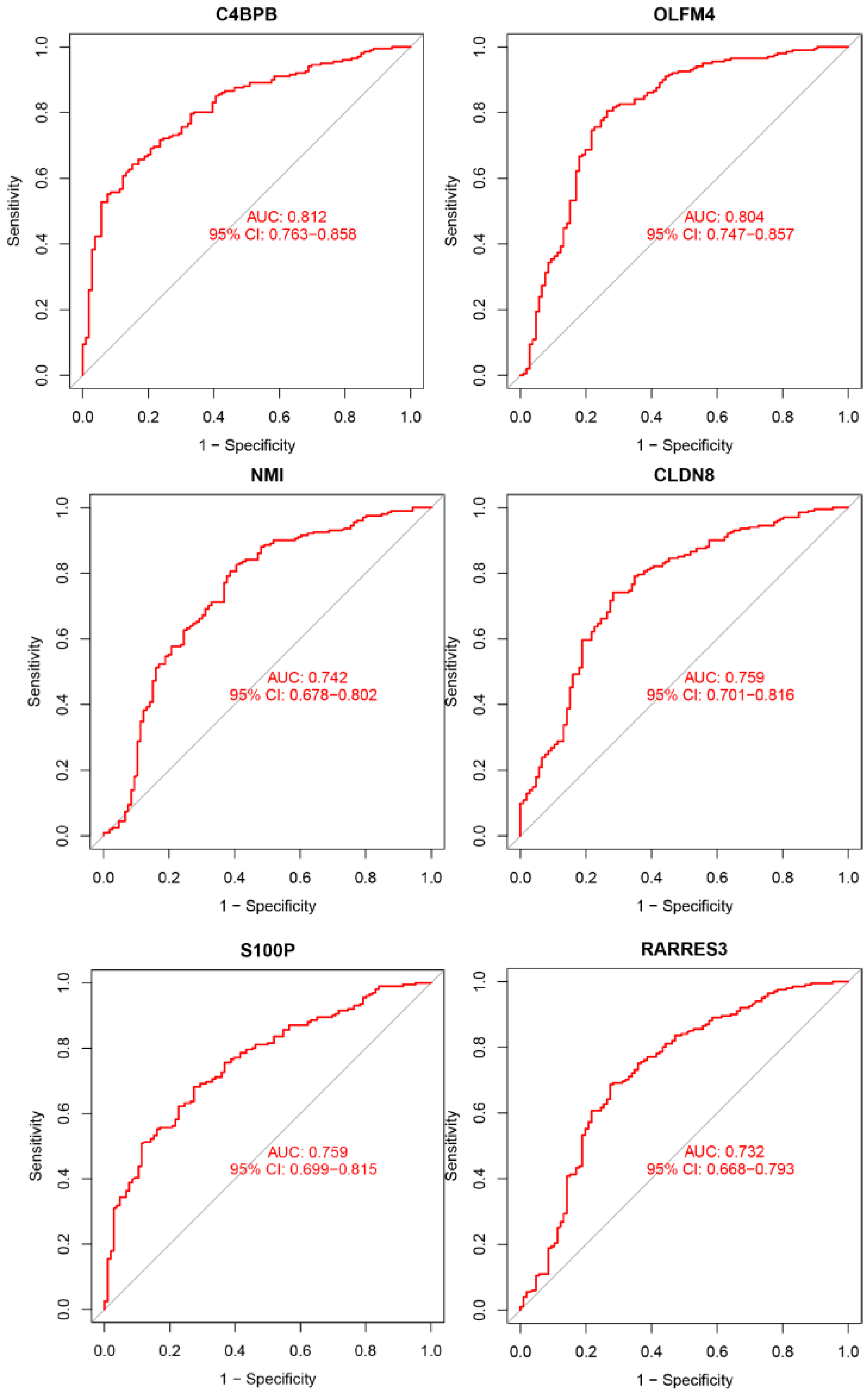

B. The ROC curve of six DGEs in testing group.

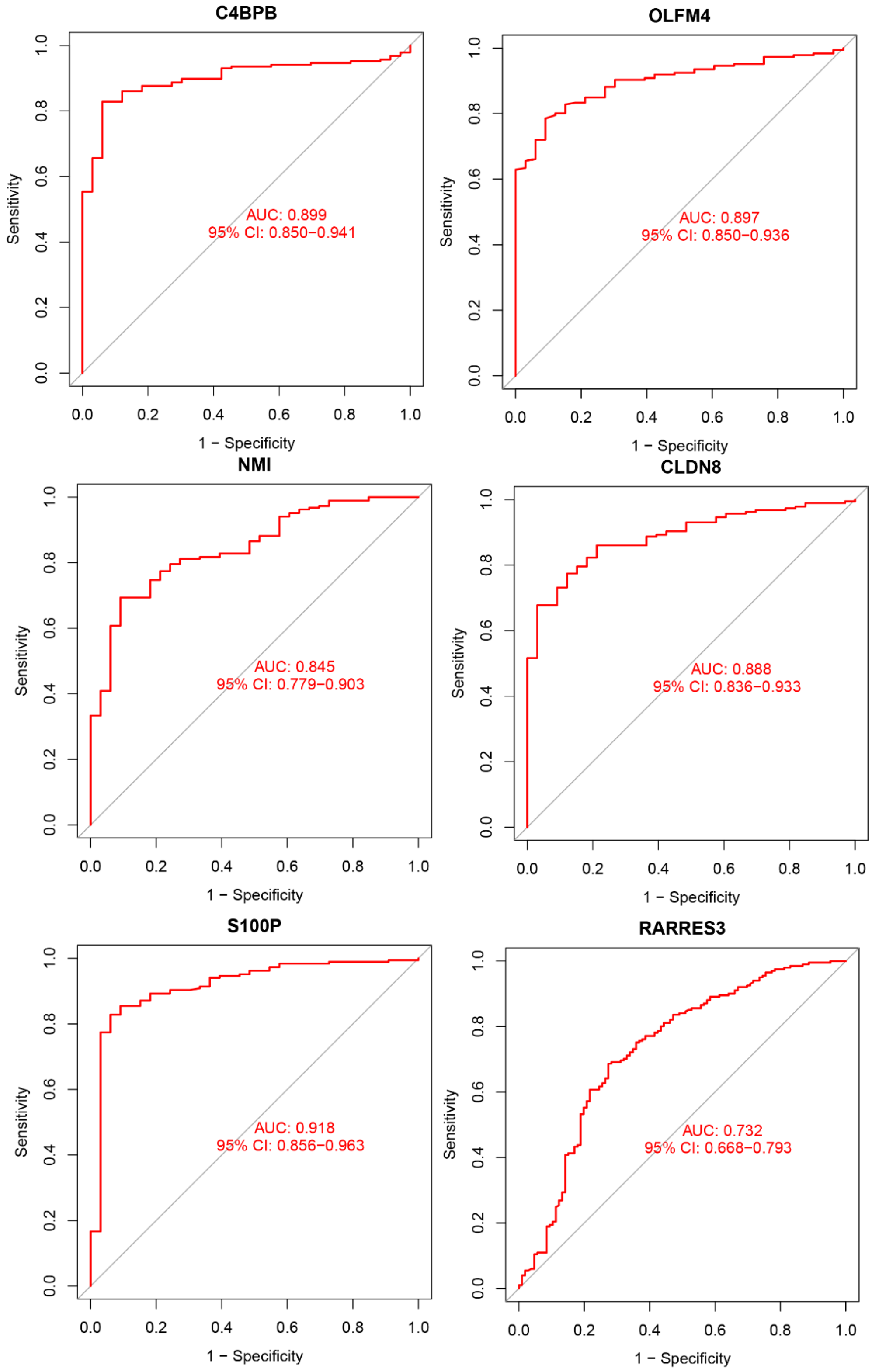

C.The ROC curve of six DGEs in GSE87473 group.

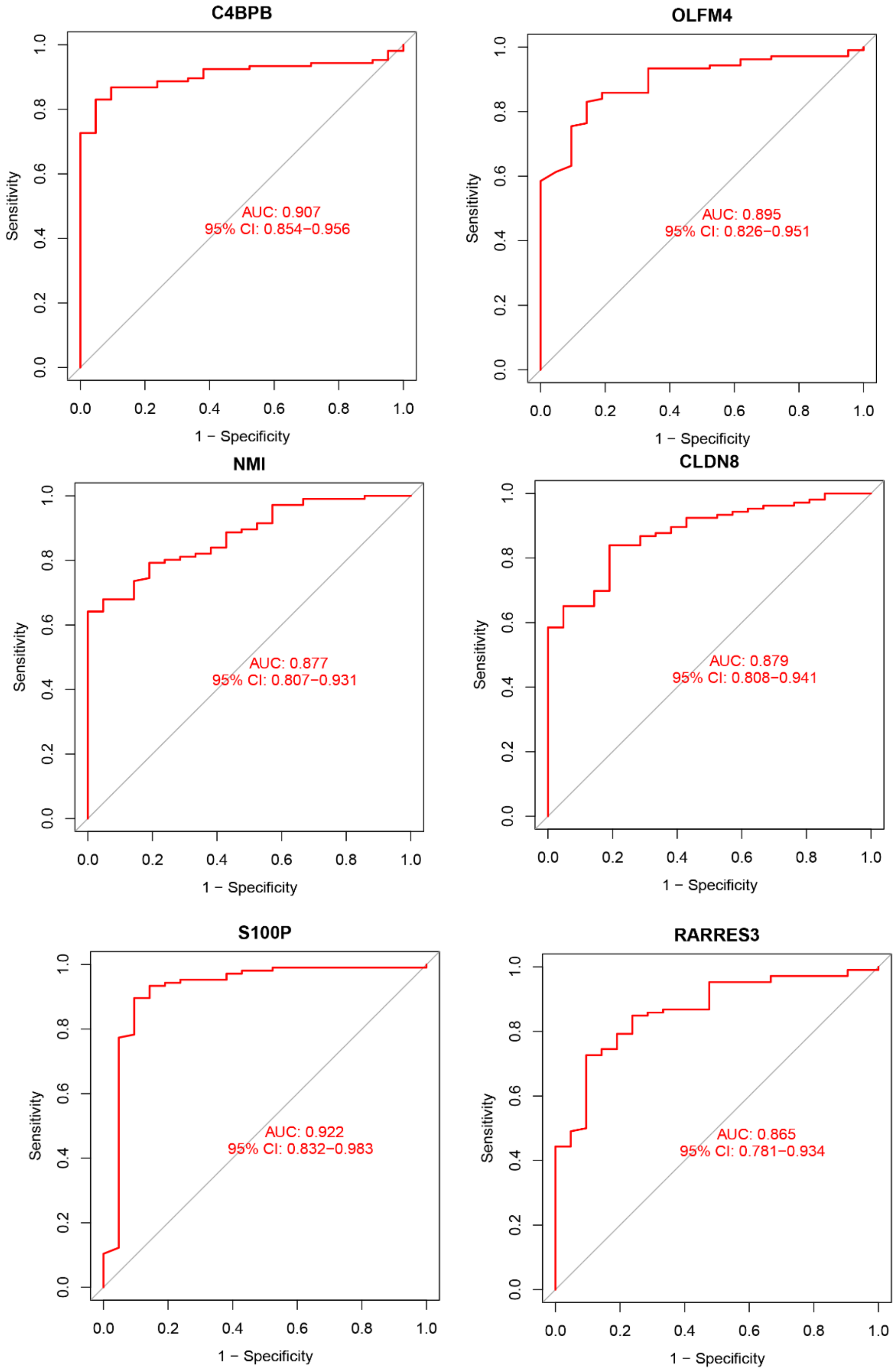

Table.3 The immune correlation in OLFM4 and C4BPB.

Gene	Cell	coef	pvalue
OLFM4	B cells naive	-0.098682612	0.106327554
OLFM4	B cells memory	0.004954595	0.935534614
OLFM4	Plasma cells	0.151136038	0.013081496
OLFM4	T cells CD8	-0.118165948	5.29E-02
OLFM4	T cells CD4 naive	0.133189367	2.90E-02
OLFM4	T cells CD4 memory resting	-0.027197559	0.656986176
OLFM4	T cells CD4 memory activated	-0.085841775	0.160334881
OLFM4	T cells follicular helper	0.026376898	6.67E-01
OLFM4	T cells regulatory (Tregs)	-0.093293088	0.126928802
OLFM4	T cells gamma delta	-0.032467321	5.96E-01
OLFM4	NK cells resting	-0.192686012	0.001496417
OLFM4	NK cells activated	0.189085373	0.001839848
OLFM4	Monocytes	0.157076371	0.009871913
OLFM4	Macrophages M0	0.222890747	2.29E-04
OLFM4	Macrophages M1	0.367895593	4.80E-10
OLFM4	Macrophages M2	-0.190324381	0.001714287
OLFM4	Dendritic cells resting	-0.088447117	0.147974807
OLFM4	Dendritic cells activated	-0.001959623	0.97447958
OLFM4	Mast cells resting	-0.13683687	0.024806174
OLFM4	Mast cells activated	0.132455403	0.029863819
OLFM4	Eosinophils	0.066298832	0.278581888
OLFM4	Neutrophils	0.102593603	0.093101747
C4BPB	B cells naive	-0.191432696	0.001608662
C4BPB	B cells memory	-0.215880883	0.000361966
C4BPB	Plasma cells	0.083547767	0.171846706
C4BPB	T cells CD8	-0.123440541	0.043084461
C4BPB	T cells CD4 naive	0.00055369	0.992788103
C4BPB	T cells CD4 memory resting	-0.067251168	0.271720323
C4BPB	T cells CD4 memory activated	0.039323204	0.520747528
C4BPB	T cells follicular helper	-0.059376462	0.331962951
C4BPB	T cells regulatory (Tregs)	-0.101394459	0.09700943
C4BPB	T cells gamma delta	-0.16062717	0.00830595
C4BPB	NK cells resting	-0.079715613	0.192431426
C4BPB	NK cells activated	0.263435637	1.20E-05
C4BPB	Monocytes	0.188622811	0.00188884
C4BPB	Macrophages M0	0.092585534	0.12984854
C4BPB	Macrophages M1	0.41247558	1.79E-12
C4BPB	Macrophages M2	-0.219571752	0.000284678
C4BPB	Dendritic cells resting	-0.027643745	0.651725753
C4BPB	Dendritic cells activated	0.10131241	0.097281501

C4BPB	Mast cells resting	-0.267854801	8.41E-06
C4BPB	Mast cells activated	0.413919247	1.47E-12
C4BPB	Eosinophils	0.062619344	0.306187078
C4BPB	Neutrophils	0.379899356	1.16E-10
